# The effect of major depression on participation in preventive health care activities

**DOI:** 10.1186/1471-2458-9-87

**Published:** 2009-03-25

**Authors:** Scott B Patten, Jeanne VA Williams, Dina H Lavorato, Michael Eliasziw

**Affiliations:** 1 Department of Community Health Sciences. University of Calgary. 3330 Hospital Drive NW, Calgary, Canada; 2 Hotchkiss Brain Institute, 3330 Hospital Drive NW, Calgary, AB, T2N 4N1, Canada

## Abstract

**Background:**

The objective of this study was to determine whether major depressive episodes (MDE) contribute to a lower rate of participation in three prevention activities: blood pressure checks, mammograms and Pap tests.

**Methods:**

The data source for this study was the Canadian National Population Health Survey (NPHS), a longitudinal study that started in 1994 and has subsequently re-interviewed its participants every two years. The NPHS included a short form version of the Composite International Diagnostic Interview (CIDI-SF) to assess past year MDE and also collected data on participation in preventive activities. Initially, we examined whether respondents with MDE in a particular year were less likely to participate in screening during that same year. In order to assess whether MDE negatively altered the pattern of participation, those successfully screened at the baseline interview in 1994 were identified and divided into cohorts depending on their MDE status. Proportional hazard models were used to quantify the effect of MDE on subsequent participation in screening.

**Results:**

No effect of MDE on participation in the three preventive activities was identified either in the cross-sectional or longitudinal analysis. Adjustment for a set of relevant covariates did not alter this result.

**Conclusion:**

Whereas MDE might be expected to reduce the frequency of participation in screening activities, no evidence for this was found in the current analysis. Since people with MDE may contact the health system more frequently, this may offset any tendency of the illness itself to reduce participation in screening.

## Background

The health belief model originated from Hochbaum's report on X-ray screening for tuberculosis [1]. Modern applications of this model in public health emphasize cognitive processes, motivation and self-efficacy [2]. Since negative cognitive style, diminished motivation and diminished self-efficacy are all clinical manifestations of major depressive episodes (MDE), the Health Belief Model leads to the hypothesis that depressive disorders would reduce participation in preventive activities. Intuition also suggests that symptoms such as hopelessness and fatigue would diminish participation. Clinical studies have reported results broadly consistent with this hypothesis [3-5] but most community-based studies of screening participation have failed to evaluate the role of depression [6-9] or have used only depressive symptom ratings [10,11]

One study conducted in a US primary care setting found no differences in the rate of mammography between women with hypertension and women diagnosed with depression [12]. Kaida et al. [13] examined determinants of Pap testing in a study that included an assessment of MDE based on a short form version of the Composite International Diagnostic Interview (CIDI-SF) [14]. An age-interaction was found in this analysis suggesting that depression may be associated with reduced testing in the 40 to 59 year age group, but depressed women 18 to 39 years old had an increased frequency of testing. In one study of military veterans, psychiatric disorders were associated with a lower level of participation in preventive activities (including mammography and Pap tests), but the specific role of MDE was not addressed in this study [15].

One study using a depressive symptom rating scale (rather than a diagnostic measure of major depression) found that high levels of depressive symptoms reduced mammography screening by a modest extent, but had no impact on Pap tests [16]. Another study of mammography participation found that people with depressive symptoms were less likely to respond to a mammography screening invitation [10]. Apparently, no studies have sought to determine whether depressive disorders may influence the receipt of blood pressure checks.

Overall, there is surprisingly little available information about the possible role of depressive disorders as a barrier to participation in screening activities in community populations. The objective of this study was to evaluate the association between MDE and participation in screening activities in a Canadian population sample. We were interested in whether reduced participation in screening was associated with depressive episodes during years when episodes occurred, and also in whether MDE disrupts ongoing screening.

## Methods

### The National Population Health Survey

The data source for this analysis was a Canadian study called the National Population Health Survey (NPHS). The NPHS is a longitudinal study based on a nationally representative community sample assembled by Statistics Canada (Canada's national statistical agency) in 1994/1995. Detailed information about NPHS methods may be found on the Statistics Canada Web page .

The target population for the NPHS consisted of household residents in the ten Canadian provinces, comprising 98% of the national population. Residents of institutions, homeless persons, people living on Indian Reserves, Crown Lands or Armed Forces Bases were excluded from the sampling frame. Some remote areas in Ontario and Quebec were also excluded. The NPHS employed a stratified two-stage sample design (clusters, dwellings) based on sampling frames developed in previous studies (a national survey called the Labour Force Survey in all provinces except Quebec and in Quebec a survey called the Enquête sociale et de santé). A respondent was then randomly sampled from the selected dwellings. To correct for design effects resulting from clustering and stratification in the sampling procedure, Statistics Canada recommends a bootstrap procedure that uses a set of 500 replicate sampling weights that they calculate and supply to researchers for this purpose.

The NPHS cohort has been interviewed every two years since the initiation of the study, in 1996, 1998, 2000, 2002 and 2004 (data from "cycle 7" collected in 2006 were not available at the time of this analysis). Available data therefore covered a ten year period between1994 and 2004. Response rates ranged from 92.8% in 1996 to 77.6% in 2004. Item non-response was generally less than 1% during follow-up. Refusal rates during follow-up ranged from 3.1% and 8.3% per cycle and between 1.7% and 5.9% per cycle were lost to follow-up because they could not be located. By the sixth cycle in 2004 (the endpoint for the current analysis), 1,680 of the original respondents were deceased, 144 had been institutionalized and 3,862 were classified as non-respondents due to loss to follow-up (refusal or failure to trace). An attrition analysis for the NPHS was reported by Beaudet et al. [17]. Attrition was found to be related to several variables, but not to MDE. This is consistent with the literature of prospective psychiatric epidemiological studies. MDE may increase loss to follow-up by mechanisms such as "unable to locate," but tends to decrease the frequency of refusal, such that the net effect on attrition is weak [18,19]. The NPHS is compliant with the Helsinki guidelines. The analysis of NPHS data reported in this paper was approved by the University of Calgary Conjoint Health Ethics Review Board.

The NPHS is a general health survey with broad topical coverage: data concerning health status, health determinants and health care utilization are collected. Interviews are conducted using Computer Assisted Interviewing procedures and carried out by well-trained and experienced interviewers. In the baseline survey approximately 75% of the interviews were conducted in-person but in subsequent cycles approximately 95% of interviews were conducted over the phone. It is not possible to report the number of items in the interview since it contains skips (e.g. the depression module contained a skip if one of two symptoms required by the DSM-IV criteria were not present) and eligibility for some questions was age and/or sex specific. However, the NPHS interviews generally lasted less than one hour.

The longitudinal cohort included 17,276 participants of any age, but the analyses reported here are restricted to relevant age and sex groups for each of the three preventive activities examined: blood pressure checks (age greater than 45 years, n = 6388), mammography (women aged 50 – 69 years, n = 1868) and pap tests (women 18 years old or older, n = 7661). Two thousand twenty two children (age < 12) were excluded from the current analysis.

### Assessment of Major Depression

The NPHS interview included the Composite International Diagnostic Interview Short Form (CIDI-SF) [14] for MD, which assesses past year major depressive episodes. This is the same instrument used in the Kaida et al. study [13]. The CIDI-SF uses a point-based scoring algorithm that incorporates the number of symptom-based criteria fulfilled and the necessity for at least one of two key symptoms (depressed mood and loss of interest or pleasure) in keeping with the Diagnostic and Statistical Manual of Mental Disorders (DSM-IV) [20]. A score of 5 on the CIDI-SF is also consistent with DSM-IV-defined MD on the basis of face validity since the manual requires fulfillment of five of nine specified symptoms, including at least one of the two key symptoms. Furthermore, this cut-point maximized performance of the CIDI-SFMD in a DSM-IIIR-based receiver operator curve analysis carried out during the instrument's development [14]. The CIDI-SFMD has produced credible prevalence estimates during applications in Canada [21,22], the US [23,24] and elsewhere [25]. In the NPHS and CCHS surveys the CIDI-SMFD has consistently replicated the expected pattern and strength of association with demographic and clinical variables [22,26-28]. Furthermore, incidence estimates from the CIDI-SFMD [27,28] are consistent with those of a systematic review by Waraich et al. [29].

### Measurement of Participation in Preventive Health Activities

Three preventive health procedures have been measured in each cycle of the NPHS: blood pressure checks, mammography and Pap tests. Notably, these are procedures for which self-report data generally agree well with medically recorded information [30]. King et al. reported 94% positive and 100% negative predictive values for self-reported mammography [31]. Tisnado et al. also reported high positive and negative predictive values for mammography (88% and 91%) and for Pap tests (93% and 85%) [32]. Inconsistency in self-reported mammography data [33] can be largely explained by problems recalling the dates, rather than whether the procedure was done [34].

NPHS items assessing preventive procedures were similar for each of the three procedures examined. In the case of blood pressure checks, the item was: "Now a few questions about your use of health care services. Have you ever had your blood pressure checked?" If the response was affirmative, this was followed by "When was the last time." Successful screening consisted of reporting each of the three procedures within the preceding year, approximately consistent with Canadian guidelines [35], see also . In the 1994 baseline NPHS interview, 84% of eligible respondents reported that they had ever had a Pap test, 60% reported ever having had a mammogram and 96% reported that they had ever had their blood pressure checked.

### Measurement of Additional Variables

Variables considered potential confounders were included in models in order to determine whether adjustment for these variables altered the observed strength of association. Selection of variables to be included in the models was based on a judgment about which variables are associated with depression and could also plausibly act as independent determinants of participation in preventive activities. The list included age, sex, rural place of residence, education level, a diagnosis of hypertension, other chronic conditions, income, medication use and employment status. Younger age, female sex, low income and unemployed status are associated with MDE in the NPHS, for example, see analyses reported by Beaudet [26,27]. Chronic conditions are also associated with MDE in the NPHS [36,37]. Some other Canadian studies have also identified weak associations between education level [38], rural place of residence [28] and MDE, although NPHS analyses have not. These variables were measured with standard items in the NPHS interview. The assessment of chronic conditions was based on an item inquiring about professionally diagnosed health conditions: "*Now I would like to ask about certain chronic health conditions which you may have. We are interested in long-term conditions that have lasted, or are expected to last, 6 months or more and that have been diagnosed by a health professional*." This was followed by a series of specific queries, for example, "*Do you have high blood pressure?" *The interview included such inquiries into 22 different chronic medical conditions.

### Analysis

We initially examined the effect of MDE on participation in preventive behaviors using cross-sectional data collected at each of the NPHS interview cycles: 1994, 1996, 1998, 2000, 2002 and 2004. Next, we used proportional hazard models to examine whether MDE leads to discontinuation of screening activities. The models were fit as generalized linear models of the binomial family with a complementary log-log link function using a non-parametric approach. Jenkins [39] outlines procedures for implementation of these analyses in STATA [40]. Models containing only MDE as a predictor (unadjusted) and those adjusting for a variety of additional variables were explored. Interactions between MDE and other covariates were explored in the multivariate analyses, but no significant interactions were found. The NPHS cohort was divided into MDE and non-MDE groups. MDE was treated as a time-varying factor. MDE status at the start of each 2-year incidence interval determined whether a respondent was in the exposed or non-exposed cohort during that interval. Respondents who were lost to follow-up, died or were institutionalized were censored in the analysis. All analyses were conducted using STATA [40].

## Results

### Blood Pressure Checks

Cross-sectionally, there was no evidence that people with MDE fail to receive blood pressure checks more frequently than those without. In fact, in several cycles the frequency with which depressed respondents went unscreened was considerably lower than that of non-depressed respondents, see Table 1.

**Table 1:** Blood Pressure Checks: Proportion of eligible NPHS respondents (n) failing to be screened in the preceding year, by NPHS cycle and Major Depression status*

	n	Major Depression % (95 CI)	No Major Depression % (95 CI)
1994	5,847	14.2 (8.0–20.3)	20.0 (18.6–21.4)
1996	5,395	9.2 (3.7–14.7)	16.0 (14.7–17.3)
1998	4,900	6.1 (1.6–10.6)	13.3 (12.0–14.6)
2000	4,247	5.6 (1.0–10.1)	10.1 (8.9–11.3)
2002	3,714	5.8 (0.8–10.9)	8.8 (7.6–10.0)

* 2004 estimates could not be released according to Statistics Canada guidelines, due to imprecision. These estimates include all eligible (age > 45) respondents irrespective of whether they had a blood pressure check at baseline.

There were 4813 respondents who reported that they had their blood pressure checked in the year preceding the baseline interview. The prevalence of MDE in this group was 4.1% (95% CI 3.5 – 4.8), comparable to the overall prevalence of MDE. Of these, n = 348 reported two years later that they had not had a blood pressure check in the year preceding that interview. The incidence of entry into an unscreened state during this initial 2-years of NPHS follow-up was 8.3% (95% CI 3.0 – 13.6) in those with MDE and 9.0% (95% CI 7.9 – 10.2) in those without MDE at the 1994 interview. The high frequency of transition from screened to unscreened status during the 1994 to 1996 interval was not sustained during subsequent follow-up. The frequency diminished from 6.3% between in the 1996 to 1998 interval to 1.9% from in the 2002 to 2004 interval.

The unadjusted hazard ratio representing the effect of MDE on the risk of transition to non-screened status was 0.84 (95% CI 0.5 – 1.4) and was not significantly different from the null value (Wald test, p = 0.53). In a proportional hazards model that included a variety of covariates (age, sex, rural place of residence, education level, a diagnosis of hypertension, other chronic conditions, income and employment status) it was found that reporting a diagnosis of high blood pressure greatly reduced the risk of discontinued screening (HR = 0.2, p < 0.001). Other variables likely to be related to health care use were also associated with a reduced risk of discontinued screening: a diagnosis of any other chronic condition (HR = 0.6, p < 0.001), female sex (HR = 0.8, p = 0.05) and age 66+ (HR = 0.6, p < 0.001). All of these variables can be regarded as potential confounders since they are associated with the transition to non-screened status. However, an adjusted HR for MDE from a model including these variables resembled the unadjusted one, and did not provide evidence of an association (HR = 0.9, 95% CI 0.5 – 1.6), p = 0.74.

### Mammograms

Table 2 presents the frequency with which respondents reported not having a mammogram during the preceding year. The estimates vary considerably from cycle to cycle and the confidence intervals are wide. This reflects diminished precision resulting from restriction of the analysis to the eligible age and sex group. Only the 2000 and 2004 results suggest a possible association, and these effects are in opposite directions. In 2000, the frequency of failed screening was higher in those with MDE, but in 2004 it was lower.

**Table 2:** Mammograms: Proportion of eligible NPHS respondents (n) failing to be screened in the preceding year, by NPHS cycle and Major Depression status*

	n	Major Depression % (95 CI)	No Major Depression % (95 CI)
1994	1,283	55.2 (39.9–70.6)	47.8 (44.2–51.4)
1996	1,304	32.9 (17.7–48.2)	47.9 (44.5–51.3)
1998	1,310	50.3 (31.7–68.9)	44.0 (40.2–47.8)
2000	1,252	24.4 (8.7–40.1)	46.8 (43.2–50.4)
2002	1,149	43.3 (22.7–63.8)	51.1 (47.5–54.8)
2004	1,107	74.4 (58.3–90.5)	51.4 (47.3–55.4)

*These estimates include all eligible respondents (women aged 50 – 69) irrespective of whether they had a mammogram in the year preceding the baseline interview.

There were 669 women who reported having a mammogram in the 12 months preceding the 1994 interview. Transition to unscreened status was defined as failure to report having a mammogram in the year preceding the next interview. This transition occurred at a frequency of 33.3% (95% CI between 1994 and 1996) and remained > 20% at each subsequent cycle. There was no evidence of an effect of age, as the frequency of discontinuation in the initial interval (1994 to 1996) was comparable in the 50–59 age category (31.2%, 95% CI 25.2 – 37.3) as in the 60–69 age category (36.3% 95% CI 28.6 – 43.9).

The unadjusted HR was not significantly elevated: HR = 1.2 (95% CI 0.8 – 1.8), Wald test, p = 0.42. In proportional hazards modeling, the same set of covariates employed in the blood pressure checks analysis (see previous section) were included, except for sex. Also, receipt of hormone replacement therapy was included as a covariate in this part of the analysis. However, none of these variables significantly predicted discontinuation of annual mammograms and their inclusion, either individually or simultaneously, resulted in no substantial change in the HR for MDE. The adjusted HR was 0.8 (95% CI 0.4 – 1.6).

### Pap Tests

Table 3 presents the frequency of failing to receive a Pap test at each of the six NPHS cycles in eligible respondents with and without MDE. Due to inclusion of a larger set of respondents, the estimates are more precise than that seen in the mammography analysis. In no instance do the point estimates or confidence intervals provide evidence of a cross-sectional association between MDE and Pap tests.

**Table 3:** Pap Tests: Proportion of eligible NPHS respondents (n) failing to be screened in the preceding year, by NPHS cycle and Major Depression status*

	n	Major Depression % (95 CI)	No Major Depression % (95 CI)
1994	6,282	42.2 (36.1–48.2)	44.8 (43.1–46.4)
1996	6,073	46.0 (38.5–53.4)	44.4 (42.5–46.3)
1998	5,850	45.4 (37.8–52.9)	46.2 (44.4–48.0)
2000	5,518	45.3 (38.5–52.2)	45.2 (43.5–46.8)
2002	5,062	50.5 (42.9–58.0)	48.3 (46.4–50.2)
2004	4,755	55.0 (47.2–62.8)	48.7 (46.8–50.6)

*These estimates include all eligible (women age 18+) respondents irrespective of whether they reported having a Pap test at the baseline interview.

There were n = 3,392 who reported having a Pap test during the year preceding the 1994 interview. By 1996, n = 902 reported not having been screened. The frequency of discontinuation in this initial follow-up period in those with MDE (30.5%, 95% CI 23.5 – 37.5) resembled that of respondents without MDE (28.9%, 95% CI 26.5 – 31.2).

The unadjusted HR over the entire follow-up interval was 1.3 (95% CI 0.9 – 1.4), which was not statistically significant (Wald test, p = 0.31). Inclusion of covariates (age group, rural residence, oral contraceptive use, hormone replacement therapy, having one or more chronic conditions, low income and current employment) did not alter the observed (lack of) association. Both age and oral contraceptive use were associated with discontinuation of screening. The HR for oral contraceptive use was 0.6, 95% CI 0.5 – 0.8). Contrary to the results reported by Kaida et al. [13], no interactions between MDE and age were identified. This may be due to the smaller sample size in the NPHS as compared to the dataset analyzed by Kaida et al. With adjustment for this set of covariates, the adjusted HR quantifying the association between MDE and failure to have a Pap test resembled the unadjusted HR, 1.2 (95% CI 0.9 – 1.5) and did not achieve statistical significance (Wald test, p = 0.14).

## Discussion

The analyses presented here failed to find evidence that MDE is an important determinant of participation in three preventive health care activities. These results are in some respects counter-intuitive since symptoms of depression would seem capable of interfering with participation in screening activities. However, there are a variety of possible explanations for these results. First, major depression is associated with a higher frequency of health care use (both for psychiatric and non-psychiatric services) [41,42]. This may result in an increased frequency of blood pressure checks, mammograms and Pap tests. Second, as MDE is often managed using medications, the need for medication renewals may also lead to increased contact with physicians. Depressive symptoms, which have been a focus of most previous studies [10,11,16], may not have been accompanied by medical interventions to the same extent as MDE, potentially explaining why some differences in screening participation have been reported in association with elevated depressive symptom levels by some prior studies.

There are several limitations of this study. First, whereas the NPHS assessed participation in three preventive activities, it was not possible to determine precisely whether the participation was for preventive purposes as opposed to treatment purposes. Another limitation is that the methods of measuring preventive activities in the NPHS did not necessarily align with levels of participation mandated by particular screening guidelines. For example, guidelines for the frequency of mammography depend on age and may include recommendations for annual or biannual screening, but only past year mammography was consistently available in the NPHS. In addition, the assessment of participation depended on self-report. Self report may be vulnerable to error either because procedures were forgotten, or because social desirability biases favor reported participation. Another issue concerns representativeness. The NPHS cohort derives from a general population sample but factors affecting continued participation during follow-up may have diminished its representativeness over time. Also, because the longitudinal components of the current analysis had the goal of clarifying temporal effects, prospective analyses were restricted to those respondents who were in the screened category at baseline. The prospective results therefore may represent an initially health-conscious group rather than in the population as a whole. Although the study included adjustment for potential confounding variables, the NPHS is a general health survey and the variables available for analysis were limited. In particular, direct measures of psychological variables relevant to screening behaviors, such as those identified by the health belief model would have been valuable to include in the analysis. The HRs for mammography and Pap tests were slightly elevated, although these elevations did not achieve statistical significance. Weak effects may exist and may not have been detected because of Type II error. They may have achieved statistical significance had the sample size been larger.

The measure of MDE used in the study, the CIDI-SF, is a brief predictive interview. It is less detailed than lengthier versions of the CIDI interview, which may have led to measurement inaccuracy in some instances. If a substantial degree of misclassification did occur, this could lead to a dilution of effect. Non-differential misclassification bias, therefore, remains an alternative explanation for the largely negative findings reported here. A related observation concerns the precision of the estimates. Although no evidence of an effect of MDE on screening participation was found, some of the confidence intervals included a range of values that may nevertheless be of public health significance. As such, the results reported here cannot entirely exclude the possibility that MDE is a determinant of screening participation.

## Conclusion

If MDE were associated with diminished participation in preventive health care activities special efforts to increase or safeguard the participation of people who experience these episodes would be advisable. However, the current analysis found no evidence for diminished participation. Increased contact with the health system, which is associated with MDE, may offset any tendency of the illness itself to reduce participation in screening.

## Competing interests

The authors declare that they have no competing interests.

## Authors' contributions

SB, and ME are the authors of a Canadian Institutes of Health Research grant supporting the project. JVAW and DHL contributed to the writing of the grant and made substantial contributions to the analysis of data and preparation of a manuscript. SB wrote the first draft of the manuscript, which was revised with input from all authors.

## Pre-publication history

The pre-publication history for this paper can be accessed here:


